# Tobacco products: Law applies also to social media

**DOI:** 10.18332/tpc/202934

**Published:** 2025-03-24

**Authors:** Christopher Heidt, Katrin Schaller

**Affiliations:** 1 Office for Cancer Prevention and WHO Collaborating Centre for Tobacco Control, German Cancer Research Center, Heidelberg, Germany

**Keywords:** prevention, advocacy, social media, tobacco products, advertising

**Dear Editor,**

Advertising for tobacco products is banned in many places in Germany, according to the Tobacco Products Act^1^. The law applies to conventional tobacco products as well as to nicotine and non-nicotine e-cigarettes, and to heated tobacco products (HTPs). It also applies to social media. As globally acting networks, several social media platforms have a voluntary self-regulation for products that are harmful to health, such as tobacco. But those regulations do not work in reality^2^. The Project ‘Media Monitoring: Advertising for Tobacco, Related Products and Alcohol on Social Media’, conducted by the German Cancer Research Center (DKFZ), shows that advertising for e-cigarettes and HTPs is still present on social media in Germany^3^. The tobacco industry is continuing its established advertising strategies for their new products in social media, creating an image of seemingly less harmful, trendy lifestyle products^4^. For example (Figure 1), British American Tobacco (BAT) advertises its Glo HTPs on Instagram with images of groups of young and fashion-conscious consumers enjoying a happy get-together, thus appealing to young people’s sense of togetherness and belonging. The tobacco sticks, which are covered by the Tobacco Products Act, are not visible in the BAT images. Instead, only the electronic devices used to heat the tobacco sticks are shown. BAT also involves Music Stars in its campaigns, using their popularity to reach a young target group. Philip Morris does things in a similar way. The tobacco company advertises the IQOS HTPs on Facebook and Instagram, sometimes even showing the tobacco sticks. In addition to the company, retailers are promoting IQOS on social media. In the media monitoring project, we collected 89 posts from retailers promoting IQOS between February and September 2023. Promotional items such as the packaging of the heating device (n=23; 26% of retailers posts for IQOS), the heating device (n=23; 26% of retailers posts for IQOS), display stands (n=6; 7% of retailers posts for IQOS) and other promotional items (n=25; 28% of retailers posts for IQOS) can be seen in the images posted. Retailers also promote the tobacco sticks (n=12; 13% of retailers posts for IQOS).

**Figure 1:**
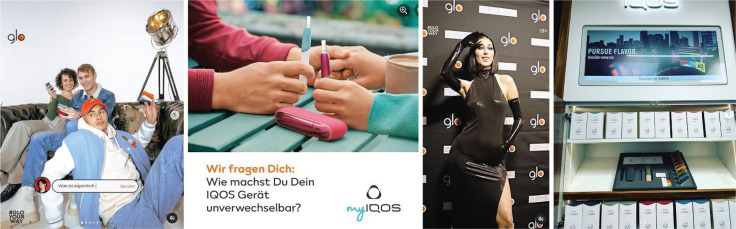
Manufactures and retailers promote HTPs on Instagram and Facebook in Germany: (from left to right) young peer group with heating devices; the heating devices with tobacco sticks (post asks, ‘We ask you: How do you make your IQOS device unique?’); influencer on promotional event; point-of-sale display stand

Advertising on social media has a huge potential to reach many young people. A national survey in 2023 concludes that nearly 60% of the German-speaking population aged 14–19 years uses Instagram every day, and about 10% Facebook^5^. Tobacco advertising increases the likelihood of adolescents to start smoking^6^ and increases overall tobacco consumption^7^. The available studies suggest that e-cigarette advertising increases the desire to try the products^8-10^. As neither HTPs nor e-cigarettes are harmless lifestyle products, young people have to be protected from advertising for these products^11,12^. The German government is therefore urged to tighten the rules on the marketing and sponsorship of tobacco and nicotine, as recommended by the WHO Framework Convention on Tobacco Control (FCTC)^13,14^. Several health and civil society organizations started a campaign committed to ensure that children and adolescents grow up in an environment that enables them to develop their personality free from alcohol and nicotine, and to make decisions that are not influenced by commercial interests^15^. The results of the media monitoring project are valuable data for advocacy for improved advertising bans and enforcement of legislation.

## Data Availability

The data are available on request from the authors.

## References

[1] Bundesministerium der Justiz. Gesetz über Tabakerzeugnisse und verwandte Erzeugnisse. Accessed March 15, 2025. https://www.gesetze-im-internet.de/tabakerzg/

[2] World Health Organization. WHO study group on tobacco product regulation: report on the scientific basis of tobacco product regulation: ninth report of a WHO study group. August 23, 2023. Accessed March 15, 2025. https://www.who.int/publications/i/item/9789240079410

[3] Heidt C, Wüllner A, Seiler J, Ouédraogo N, Schaller K. Advertising of tobacco and related products on social media in Germany. Tob Prev Cessat. 2024;10:10.18332/tpc/195499. doi:10.18332/tpc/195499 PMC11582914 39583885

[4] World Health Organization. Heated tobacco products: summary of research and evidence of health impacts. WHO; 2023. Accessed March 15, 2025. https://www.who.int/publications/i/item/9789240042490

[5] Koch W. Ergebnisse der ARD/ZDF-Onlinestudie 2023: Soziale Medien werden 30 Minuten am Tag genutzt – Instagram ist die Plattform Nummer eins. Media Perspektiven; 2023. Accessed March 15, 2025. https://www.ard-media.de/media-perspektiven/publikationsarchiv/2023/detailseite-2023/ard-zdf-onlinestudie-2023-soziale-medien-werden-30-minuten-am-tag-genutzt-instagram-ist-die-plattform-nummer-eins-2-1-1-1

[6] Lovato C, Linn G, Stead LF, Best A. Impact of tobacco advertising and promotion on increasing adolescent smoking behaviours. Cochrane Database Syst Rev. 2003;(4):CD003439. doi:10.1002/14651858.CD003439 14583977

[7] Davis RM, Gilpin EA, Loken B et al. NCI Tobacco Control Monograph Series. 19 The Role of the Media in Promoting and Reducing Tobacco Use. National Cancer Institute; 2008. Accessed March 15, 2025. https://cancercontrol.cancer.gov/sites/default/files/2020-08/m19_complete.pdf

[8] Schaller K, Kahnert S, Graen L, Mons U, Ouédraogo N. Tabakatlas Deutschland 2020. Deutsches Krebsforschungszentrum; 2020. Accessed March 15, 2025. https://www.dkfz.de/fileadmin/user_upload/Krebspraevention/Download/pdf/Buecher_und_Berichte/2020_Tabakatlas-Deutschland-2020.pdf

[9] Deutsches Krebsforschungszentrum. Werbung verführt zum Rauchen – umfassendes Tabakwerbeverbot ist überfällig. Aus der Wissenschaft – für die Politik. Deutsches Krebsforschungszentrum; 2020. Accessed March 15, 2025. https://www.dkfz.de/fileadmin/user_upload/Krebspraevention/Download/pdf/AdWfdP/AdWfdP_2020_Werbung-verfuehrt-zum-Rauchen.pdf

[10] Collins L, Glasser AM, Abudayyeh H, Pearson JL, Villanti AC. E-Cigarette Marketing and Communication: How E-Cigarette Companies Market E-Cigarettes and the Public Engages with E-cigarette Information. Nicotine Tob Res. 2019;21(1):14-24. doi:10.1093/ntr/ntx284 29315420 PMC6610165

[11] World Health Organization. Electronic nicotine and non-nicotine delivery systems: a brief. WHO. September 30, 2020. Accessed March 15, 2025. https://www.who.int/europe/publications/i/item/WHO-EURO-2020-4572-44335-62638

[12] World Health Organization. Heated tobacco products: a brief. WHO. September 30, 2020. Accessed March 15, 2025. https://www.who.int/europe/publications/i/item/WHO-EURO-2020-4571-44334-62636

[13] WHO Framework Convention on Tobacco Control. FCTC/COP/10/7 Progress Report on Technical Matters Related to Articles 9 and 10 of the WHO FCTC (Regulation of Contents and Disclosure of Tobacco Products, Including Waterpipe, Smokeless Tobacco, and Heated Tobacco Products). WHO; 2023. Accessed March 15, 2025. https://fctc.who.int/resources/publications/i/item/fctc-cop-10-7-progress-report-on-technical-matters-related-to-articles-9-and-10-of-the-who-fctc-(regulation-of-contents-and-disclosure-of-tobacco-products-including-waterpipe-smokeless-tobacco-and-heated-tobacco-products

[14] WHO Framework Convention on Tobacco Control. FCTC/COP/10/8 Tobacco advertising, promotion and sponsorship: depiction of tobacco in entertainment media. May 19, 2023. Accessed March 15, 2025. https://fctc.who.int/resources/publications/i/item/fctc-cop-10-8-tobacco-advertising-promotion-and-sponsorship-depiction-of-tobacco-in-entertainment-media

[15] Kinder ohne Alkohol und Nikotin. Initiative für den Schutz von Kindern und Jugendlichen vor Alkohol- und Nikotin-Marketing. Kinder;2024. Accessed March 15, 2025. https://kinder-ohne-alkohol-und-nikotin.de/

